# Dentoalveolar fracture: A complication of extraction of upper left first molar

**DOI:** 10.1002/ccr3.1814

**Published:** 2018-09-19

**Authors:** Zhuo Wei Tay, Sarah Sabrina Zakaria, Ahmad Khairuddin Zamhari, Sie Wei Lee

**Affiliations:** ^1^ Sarawak General Hospital Ministry of Health Kuching Sarawak Malaysia

**Keywords:** dental extraction, dentoalveolar fracture, maxillary tuberosity fracture

## Abstract

The current trend of managing maxillary tuberosity fractures is traumatic and results in the loss of bone and teeth. Treatment options that chose to retain the fractured segments and teeth have been perceived to be unfavorable. This case report shows that maxillary tuberosity fractures can be treated conservatively.

## 1 INTRODUCTION

Current trends in the management of maxillary tuberosity fracture are to remove the fracture segments along with the associated teeth. This article presents a case of a large dentoalveolar fracture during dental extraction of the left maxillary first molar. It also details the subsequent conservative management and treatment outcome.

Dental extraction is a routine dental procedure done to remove unwanted or unsalvageable dentition. It is performed using dental extraction forceps which helps the practitioner to grasp the tooth, apply the force and leverage needed to expand the alveolus, separate the periodontal ligament, and deliver the tooth. Among the “complications of dental extraction include swelling, bruising and in severe cases, fractures of the alveolar bone.^1^^”^

Dentoalveolar fracture is a fracture of the facial bones that involves a segment of the alveolus as well as the associated teeth in that segment. Fractures of this kind can be easily identified through clinical findings characteristic of this phenomenon. “Such findings include segment mobility and dislocation of several teeth as well as occlusal changes due to misalignment of the fractured alveolar segment.^2^”

“Fractures of the maxillary tuberosity is of great concern as the maxillary tuberosity is vital towards the stability of maxillary dentures.^3^” “The ideal therapeutic goal of management of maxillary tuberosity fracture is to salvage the fractured bone and to fix it in place and provide the best environment for healing.^4^” “However, due to certain situations such as small fracture segments or presence of symptomatic teeth, this approach may not always be feasible and it becomes necessary to remove the fracture segment along with the involved teeth.^5^”

Complications of tooth extractions are well‐documented in scientific literature but only a few reports are available regarding maxillary tuberosity fractures. The purpose of this paper is to present a case of a large maxillary tuberosity fracture that occurred during routine dental extraction of a maxillary left first molar as this incident has a high possibility of occurring in daily dental practice but the conservative management of such complications are rare in scientific literature.

## 2 CASE PRESENTATION

A 25‐year‐old male was referred from a primary care facility for the management of dentoalveolar fracture involving the left maxillary tuberosity during attempted extraction of maxillary left first molar. He had no known medical problems and no known allergies.

Upon examination, he presented with facial asymmetry with swelling occurring on his left face. The swelling was diffuse and slightly tender to palpation, involving the entire left buccal region from zygomatic arch to the border of the mandible. There was no limitation of mouth opening and no deviation of the mandible upon opening and closing of the mouth.

**Figure 1:**
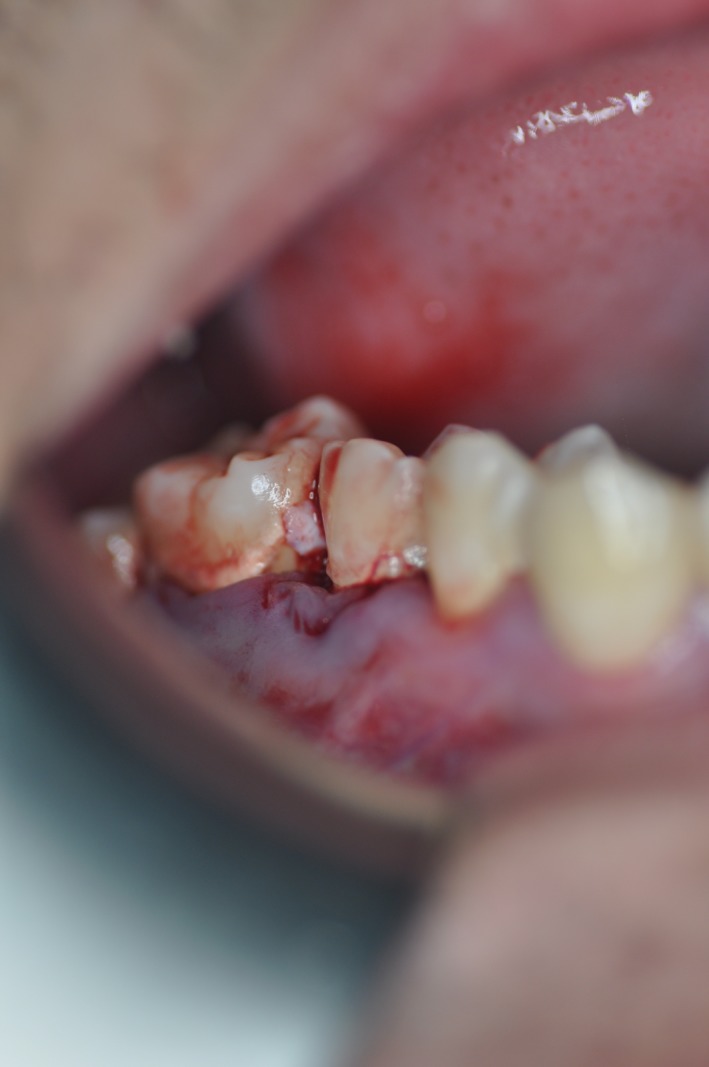
Preoperative photograph showing extruded left maxillary first molar

Intraorally, there was a mobile fracture segment seen on his left maxilla involving the left maxillary first, second, third molar and maxillary tuberosity. The segment was extremely mobile and extruded preventing full occlusion of his teeth. The maxillary left second premolar was firm. The maxillary left first molar had a large occlusal caries which extends subgingivally and was reported to be tender to percussion prior to the attempted extraction. There was a small laceration wound on the buccal gingiva adjacent to the upper left first molar measuring about 6 mm (Figure 1).

As the diagnosis as well as the extent of the dentoalveolar fracture was able to be determined clinically, no radiographical examination was done for this patient.

As the patient was a young healthy male, and the maxillary second and third molar that was involved in the maxillary tuberosity fracture was sound, the authors decided on a conservative approach to reduce and stabilize the fractured segment followed by transalveolar extraction of the unrestorable maxillary first molar at a later date. As the patient was seen toward the end of the working day, the fracture was first stabilized using eyelet wiring and an appointment was set for the following day.

During his next appointment, closed reduction and fixation was achieved using upper and lower arch bars with intermaxillary fixation (IMF). At the end of this visit, occlusion was reachieved and the fracture segment was firm. The arch bars and IMF were left in situ for a period of 4 weeks to allow for healing of the fracture. The patient was placed on an antibiotic regimen of amoxicillin and metronidazole for 1 week to prevent infection as the upper left first molar had a large occlusal caries and was reported to be tender to percussion prior to the extraction. He was reviewed weekly to assess healing and to observe for signs of infection.

During the review on the fourth week, the IMF was removed to assess the healing of the fracture and the stability of the occlusion. Some minor mobility of the fracture was noted but was deemed acceptable. The arch bars were kept in‐situ for one more week should the need to replace the IMF arise.

**Figure 2:**
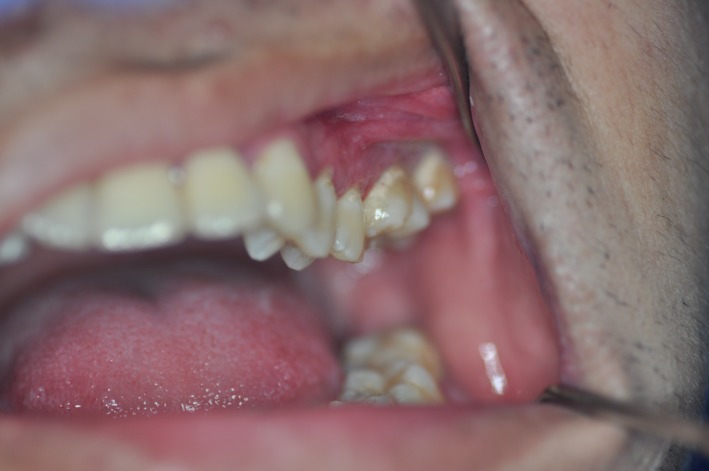
Postoperative photograph showing left maxillary first molar in original position

Upon review on the fifth week, the fracture segment was firm and occlusion was stable. There were no signs of active infection. The upper and lower arch bars were removed and a date for surgical removal of the upper left first molar was set for 1 month later (Figure 2).

The carious upper left first molar was removed via surgical transalveolar approach with no complications.

## 3 DISCUSSION

There are many etiological factors responsible for maxillary tuberosity fracture. “These includ large maxillary sinuses with thin walls, teeth with abnormally long or divergent roots or having an abnormal number of roots, ankylosis, and hypercementosis of the tooth. The presence of chronic infection of the periapical region may result in sclerosis of the bone making it more liable to fracture.^3^” In addition to these local factors, patient's systemic factors may contribute as well. Patients with lower bone density due to osteoporosis or due to certain medications or lifestyle habits can make them more susceptible to dentoalveolar fractures.

Extrinsic factors can also play a role in causing maxillary tuberosity fractures. “The application of excessive forces or inadequate alveolar support with the non‐operative hand during extraction by the clinician can result in intraoperative fracture of the maxillary tuberosity.^5^, ^6^” The use of the wrong or worn down instruments can also contribute to complications.

Dental extractions should be approached using the appropriate instruments, held in a way that ensure good control of the forces applied to the patient. “In the extraction of maxillary molars, due to the morphology and number of the roots, large initial forces should be avoided. Instead, apical forces should be applied first followed by slow and deliberate forces in the buccal and palatal direction allowing initial expansion of the surrounding alveolar bone. The buccal and palatal forces should then be slowly increased to facilitate removal of the tooth.^7^” It is imperative that the practitioner should always use controlled forces when performing dental extraction to avoid complications such as dentoalveolar fractures. Surgical removal should be considered if normal amounts of force are insufficient to deliver the tooth. The use of radiographs can aid in the treatment planning as well as determining the need for surgical removal of any teeth.

There is currently no standard procedure for the management of maxillary tuberosity fractures. “Some practitioners believe that if the fractured tuberosity is small with a tooth or two teeth, or if the tooth is infected or symptomatic at the time of removal, then the only course is to remove the teeth along with the tuberosity. They justify that the symptoms of the tooth will continue and the presence of infection will prevent full recovery of the fracture.^3^, ^5^” “Others advocate a more conservative approach of large maxillary tuberosity fractures where the tooth is gripped with extraction forceps and a sharp elevator is used to separate the tooth from the alveolar bone enabling the delivery of the tooth while keeping the tuberosity in situ.^8^” “Other practitioners suggest an even more conservative approach where the fracture segment in first stabilised using rigid fixation techniques along with adjuvant antibiotics and when the fracture is healed, the tooth in question is managed endodontically or like in our case, removed surgically.^6^”

In conclusion, fractures of the maxillary tuberosity are managed in a case by case basis. The extreme variables in terms of size of the fracture, mobility of the fracture segments, clinical status of the tooth intended to be extracted, other involved teeth and other systemic factors such as the patient's general health and well‐being as well as his lifestyle habits make forming a standardized management for tuberosity fractures a near impossibility.

We defend the conservative approach that the fractured tuberosity should be salvaged when possible, especially if the other involved dentition is still sound. Despite having a symptomatic tooth at the time of the fracture, the patient made a successful recovery with none of the expected complaints, and he retained the function of his left maxillary second and third molars which would not have been the case if the authors had removed the fractured maxillary tuberosity segment.

## AUTHOR CONTRIBUTION

All the authors contributed to the management of the patient, documentation of the case, review of all relevant literature, and the writing of the manuscript.
